# Biomechanical Aspects of Lower Limb Torsional Deformation Correction with the Ilizarov External Fixator

**DOI:** 10.1007/s10439-013-0911-6

**Published:** 2013-09-13

**Authors:** Piotr Morasiewicz, Jarosław Filipiak, Krzysztof Krysztoforski, Szymon Dragan

**Affiliations:** 1 Department and Clinic of Orthopaedic and Traumatologic Surgery, Wrocław Medical University, ul. Borowska 213, 50-556 Wroclaw, Poland; 2 Division of Biomedical Engineering and Experimental Mechanics, Wrocław University of Technology, Wroclaw, Poland

**Keywords:** Torsional deformation, Ilizarov external fixator, Kinematics of bone

## Abstract

The correction of torsional deformities with the Ilizarov apparatus is accompanied by rotational and translational displacement, which affects the biomechanics of the bone fragments. Understanding the biomechanical factors will assist in designing the optimal treatment strategy and mechanical properties of the fixator, thus shortening the duration of treatment and improving the outcomes. In order to determine the impact of different types of derotators on the kinematics of bone fragments in Ilizarov apparatus, physical models were studied. Translational and derotational displacement was measured using non-contact method (Optotrak Certus Motion Capture System). The results of the studies conducted on physical models have shown that regardless of the type of the derotator, the divergence between the applied angle of derotation and the obtained angle of rotation relative to fragments needs to be taken into account. Transverse displacement of fragments occur by 3.5 mm to approximately 9 mm, depending on the angle of derotation. For correction of rotational deformities up to 30°, it is advisable to use the type *Z* derotators because of its higher accuracy of derotation. Different types of derotators can affect the biomechanical conditions in the regenerating bone tissue through different kinematics characteristics.

## Introduction

The Ilizarov method is used in torsional deformation correction. Ilizarov fixator allows to correct external or internal torsion of the thigh and lower leg after corticotomy performance.^2^,^10^,^12^ In the context of orthopaedics, the term derotation refers to the correction of a torsional bone deformity by rotating fragments of the bone into correct orientations. A special mechanism, constructed for derotation of bone fragment, added to the Ilizarov apparatus is named derotator. Most commonly, three types of derotators: *Z* type, *H* type, and translational-derotational mechanism (TR type), are used in the Ilizarov apparatus.^2^,^13^,^16^

Internal forces originating from the response of the soft tissue surrounding the treated bone and exterior forces resulting from the influence of the gravitational field act on the complex biomechanical system created by assembling the Ilizarov fixator onto the limb segment.^1^,^5^ Adding derotators to the structure of the Ilizarov apparatus changes the fixator stiffness.^13^ Correction of torsional deformation is accompanied by rotational and translational displacement of bone fragments,^8^ resulting in a more complex biomechanics and includes the additional tension stresses, which may cause further damage to the nutritional blood supply to the bone.^14^ In the case of torsional deformation correction, precise assembly of the Ilizarov apparatus in a manner enabling the bone axis to pass as closely as possible to the centre of the ring will minimizes the translational displacement of bone fragments.^8^,^15^ Therefore, while correcting the torsional deformation with the Ilizarov method, it is advisable to use derotational mechanism that reduces adverse rotational and translational displacement of bone fragments.

Different types of derotators can affect derotational biomechanical properties of the Ilizarov apparatus within the region of the femoral segment through changes in the fixator stiffness.^13^

It can be concluded that understanding the biomechanical factors affecting the course and results of torsional deformation treatment using the Ilizarov apparatus will help in planning the optimal treatment strategy and designing modifications; thus, resulting in shortening the duration of treatment and achieving improved treatment outcomes.

In international literature we have found only one biomechanical paper concerning the treatment of torsional disorders using the Ilizarov method.^13^ There are no papers evaluating the kinematics of bone during torsional deformation treatment using the Ilizarov fixator.

The aim of our study was to evaluate the kinematics at the interfragmentary site associated with torsional deformation correction using an Ilizarov external fixator. Analysis of the biomechanical aspects of the derotation was measured using non-contact motion capture systems in physical models. The influence of derotator type and derotation range on translational and rotational displacements of bone fragments and the differences between the desired and the achieved interfragmentary derotation have been measured in a physical model.

## Materials and Methods

**Figure 1:**
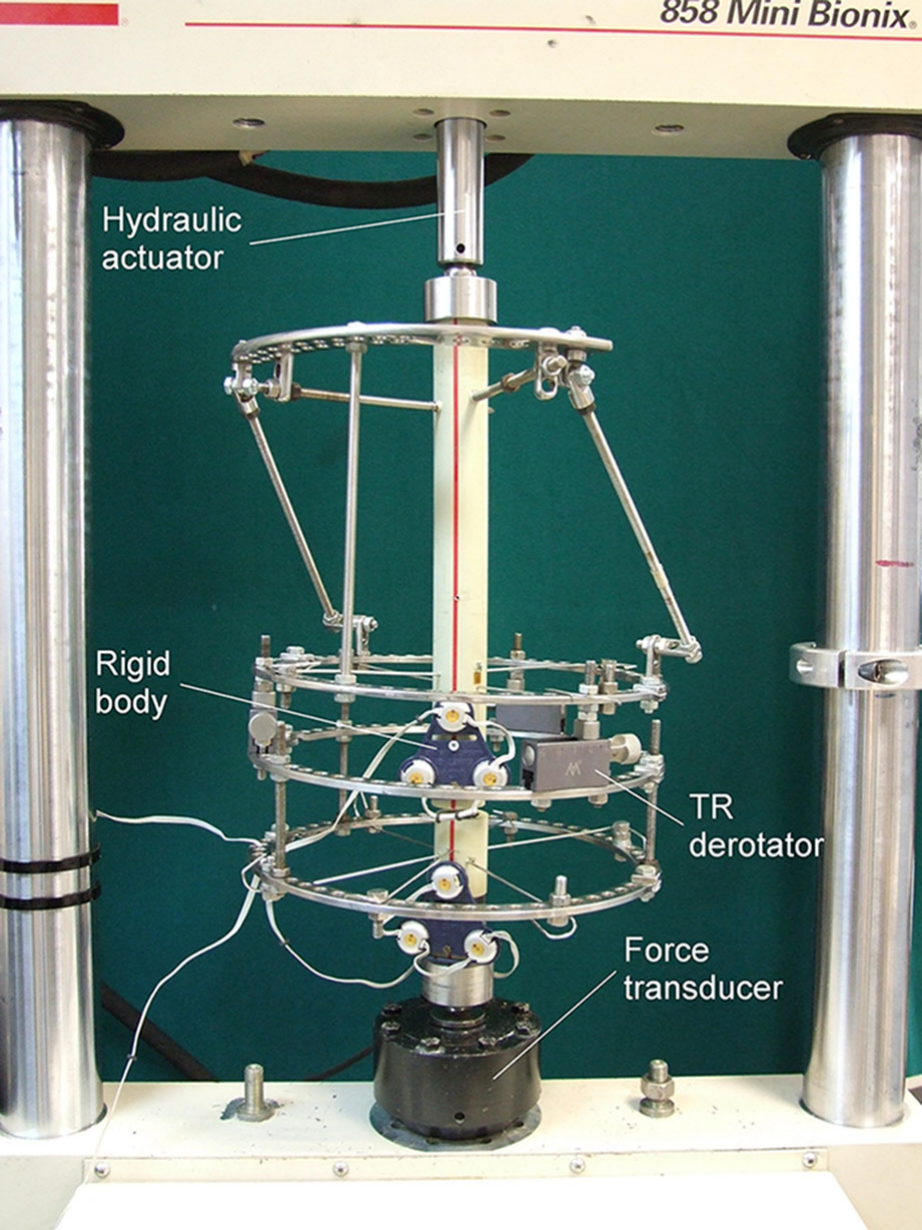
Ilizarov external fixator installed in the loading system

**Figure 2:**
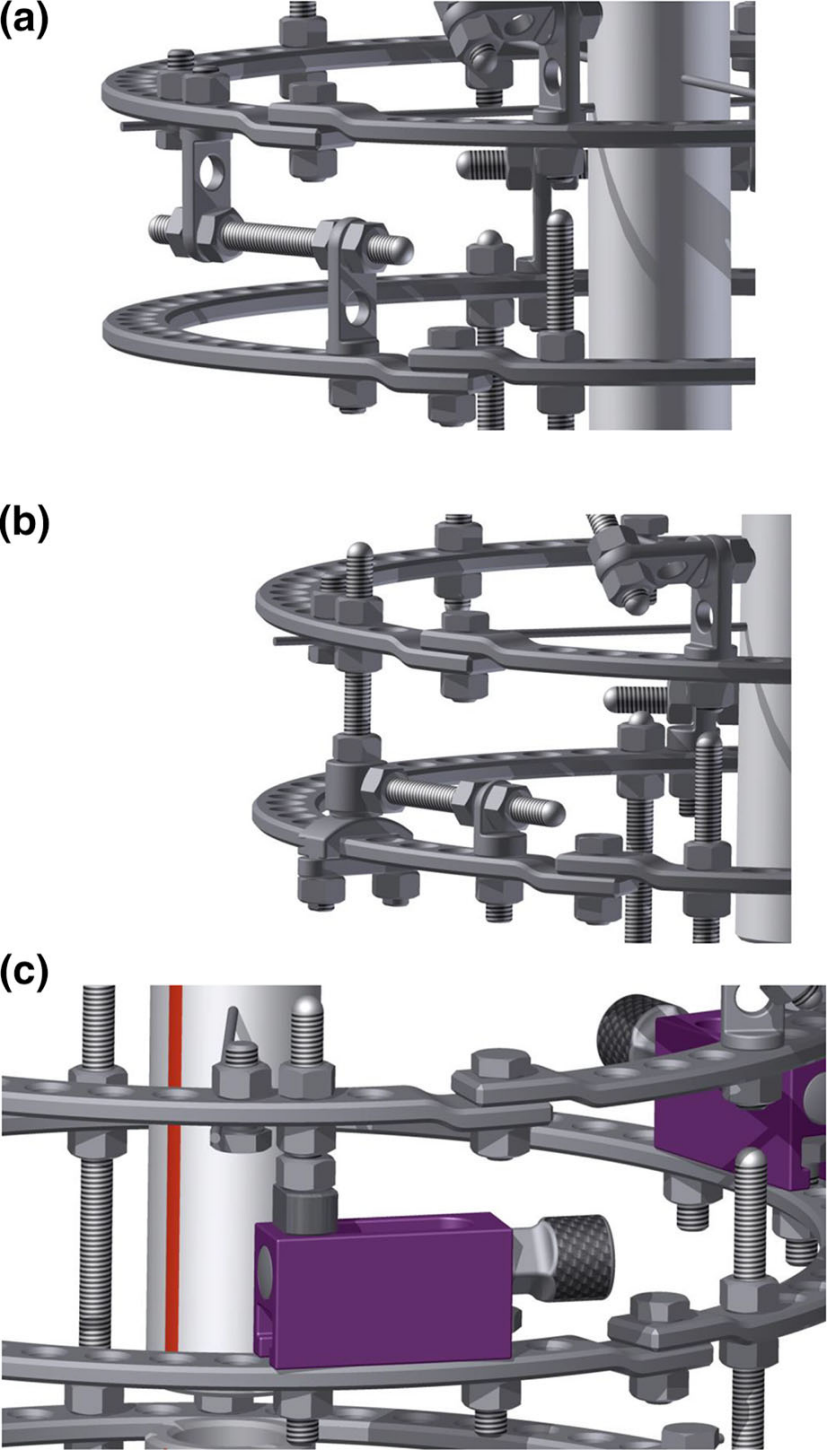
Analyzed structures of the derotators type: (a) type *Z*, (b) type *H*, (c) TR-type translational-derotational mechanism

The influence of the type of derotator included in the structure of Ilizarov fixator on the kinematics of bone fragments was analysed on the basis of experimental studies. The study was conducted on physical models, in which Ilizarov fixator with built-in derotator was mounted on polyethylene tube elements, which constituted the models of bone fragments (Fig. 1). “Osteotomy” was performed in the distal femur and the length ratios of the fragments were adapted as 3:1, which is characteristic for a case of limb derotating at the level of the femoral segment in the distal part. The design of the fixator corresponded to the typical configuration used for derotation of the femur with the hybrid system of implants (proximal arch, fixed to the intertrochanteric area with 2 Schanz screws proximal ring, fixed with 2 Kirschner wires, free middle ring, and distal ring, fixed with 3 Kirschner wires). The derotational mechanisms were installed between the middle and proximal rings. During the test, the values of angular and linear displacement of bone fragments in the horizontal plane, resulting from the derotation of the fragments, were determined by using one of three types of derotators. The study was examined *Z*-type (Fig. 2a) and *H*-type derotators (Fig. 2b), as well as TR-type translational-derotational mechanism (Fig. 2c). The *Z*-type derotation mechanism (Fig. 2a), is built of a transverse threaded rod placed parallel to the rings and two vertical two-hole male connectors, of which one is fixed to the upper ring, and the other to the lower ring. Derotation of bone fragment by *Z*-type derotator is performed by loosening one nut by two-hole male connectors and tightening the other nut with two-hole male connectors. Derotation requires loosening the nuts connecting two-hole male connectors with the rings. The second derotation mechanism, the type *H* (Fig. 2b), consists of a threaded rod placed vertically, whose one end is connected perpendicularly to the free ring, while the other end is terminated with a slider enclosing the distal ring. On the rod there is a sleeve (bushing) that can rotate around its axis. The sleeve is connected to a threaded rod running parallel to the plane of the rings. Its other end is fixed to a one-hole male connector attached to the distal ring. Derotation of bone fragment by *H*-type derotator is performed by loosening one nut with one-hole male connectors and tightening second nut by one-hole male connectors. Derotation requires loosening the nuts connecting one-hole male connectors and slider with the rings. The TR-type translational-derotational mechanism (Fig. 2c) consists of a rectangular enclosure made of aluminium alloy, with a hollow oval opening. Inside it there is a screw mechanism. The dial-powered mechanism provides smooth control of the angular displacement of adjacent rings connected with the derotator.

The tested physical models were placed in the load cell of MiniBionix 858 (MTS Systems Corp., USA). When supported, both fragments had 3° of freedom (rotations relative to three axes), while their free ends, constituting the edges of the inter-fragmentary fractured site, had the possibility of translation in the directions contained in the horizontal plane. Initially, the model was loaded with tensile axial force of 200 N. This value corresponds to the strength of the soft tissue response during elongation of the femur equal to 35–40 mm. After applying the pre-load, the derotation of the fragments by the applied angle was carried out. In the case of *Z*- and *H*-type derotators, their geometry changed (increased length of transverse threaded rod by ∆*L*), which theoretically should generate relative rotation of fragments by 15° and 30°. In the case of TR-type derotator, due to structural limitations, only derotation by 15° was performed. Derotation angles of 15 and 30° were tested, which is a typical derotation range achieved in one step treatment.^10^,^12^–^14^ The value of ∆*L* needed to generate a specific angle of rotation was determined based on the following relationship:

$$ \Updelta L  =  \alpha  \times  r, $$

where *α* represents the angle of rotation expressed in [rad], *r* is the radius of the circle, on the circumference of which we can find ∆*L* (in our case *r* = 107.5 mm and constitutes the radius of the ring coupled to the derotator).

**Figure 3:**
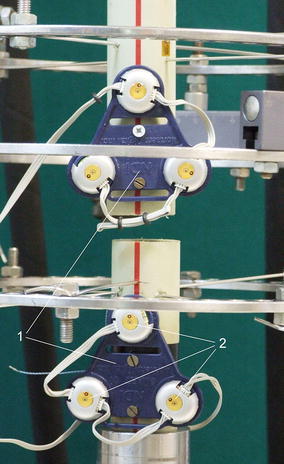
View of “rigid body” (non-contact motion capture systems) fixed to the bone fragments—polymer tube: 1—rigid body, 2—infrared diodes

Translational and rotational displacements were measured using Optotrak Certus Motion Capture System (Northern Digital Inc., Canada). Optotrak enables a non-contact 3D measurement of the position of objects in real time. This system measures the position with an accuracy of −0.15 mm in any dimension. Markers consist of infrared diodes (Fig. 3). They are placed on the examined object, and allow specifying its location in space. Submitting at least three markers non-collinearly results in the so-called “rigid body” tracked with 6° of freedom (3 translations and 3 rotations in the Cartesian reference system). Fixator with the mounted “rigid body” is shown in Fig. 1. The applied configuration of markers allowed for determining the relative displacement of the two fragments. The system applied in the study consisted of localizer recording the location of the markers and the “rigid body”, the main collecting unit for synchronizing markers and sending data to the computer, an infrared transmitter connected to the main unit for synchronizing wireless markers, as well as wireless strober with connected markers.

Five independent measures of range on translational and rotational displacements and the achieved derotation were taken for each derotator configuration of the analysed “fixator-tubular elements” system of the physical models. Altogether there were 25 tests taken in five derotator configuration. Five tests for *Z*-type derotators with relative rotation of fragments by 15°, five tests for *Z*-type derotators with relative rotation of fragments by 30°. Five tests for *H*-type derotators with relative rotation of fragments by 15°, five tests for *H*-type derotators with relative rotation of fragments by 30° and five tests for TR-type derotators relative rotation of fragments by 15°. Five tests for each derotator configuration were repeated in equal conditions. The data was analyzed using an analysis of variance (ANOVA) followed by a *post hoc* test determining the individual differences. The Student’s *t* test was applied to compare the transverse displacement and angle of rotation values between two specific configurations. The level of significance was considered to be *p* < 0.05.

## Results

The experiments were repeated five times (*N* = 5) for each of the derotators configurations. The mean results of the transverse displacement *D*_T_ and the angle of rotation *α*_R_ of bone fragments determined in physical models are presented in Table 1. Calculated derotation angles and the vector of displacement in the horizontal plane constitute the relative values for proximal and distal fragments. The research conducted on the physical models has shown that the tested derotators perform assigned derotation with varying accuracy (Table 1). TR-type derotator was found to be the most accurate in the range of up to 15°—the difference in the angle relative to the rotation of fragments amounted to +0.4°. For *Z*-type derotator the difference was −1.1°, while for *H*-type derotator it amounted to −2.4°. The differences of the derotator configurations were found to be statistically significant (*p* = 0.024). At the higher range of up to 30°, the difference for *Z*-type derotator was −3.3°, while for *H*-type derotator it amounted to −4.9°. In this case the differences of the derotator configurations were found to be statistically significant (*p* = 0.037). During measurements, translational displacements of the fragments were registered in addition to rotational displacements. The values of these movements ranged between 3.5 mm and 5.4 mm in the case of the assumed 15° derotation. When the angle of derotation was 30°, the transverse displacement already reached values between 7.6 mm (*H*-type derotator) and 8.9 mm (*Z*-type derotator). At 15° derotation, the differences of the derotator configurations were found to be statistically significant (*p* = 0.024) whereas the translational displacements differences for the 30° derotation were not significant (*p* = 0.072).

**Table 1:** Mean values of rotations and displacements of bone fragments calculated for fixators with the compared derotators configurations

Derotator configuration	Assumed nominal derotation *α* [°]	Factual derotation *α*_R_ [°] (SD)	Transverse displacement of fragments *D*_T_ [mm] (SD)
*Z* 15	15	13.9 (0.71)	3.9 (0.32)
*Z* 30	30	26.7 (0.95)	8.9 (0.21)
*H* 15	15	12.6 (0.41)	3.5 (0.47)
*H* 30	30	25.1 (0.63)	7.6 (0.35)
TR 15	15	15.4 (1.14)	5.4 (0.43)

Standard deviation (SD) in brackets

## Discussion

The use of the Ilizarov apparatus allows for the choice between gradual, mixed, or acute corrections. Lobst recommends rapid deformation correction.^11^ However, most researchers recommend gradual correction of deformation.^9^,^14^,^15^,^17^ According to Bor *et al*.,^2^ gradual deformation correction is indicated especially in the case of large and complex deformation, minimizes the risk of damage to the soft tissues, especially the nerves and blood vessels. Velazquez noted an increase in complications in the case of using the apparatus for a longer period of time and comprehensive treatment.^18^ Rapid correction reduces the duration of fixator use, which potentially minimizes the risk of complications associated with long-term retention of fixator and reduces the cost of treatment. Reduction of the treatment period increased patient comfort.^7^

Current problem in the Ilizarov method is a better control of bone fragment during the treatment process.^7^ There is no study evaluating the kinematics of bone during torsional deformation correction using the Ilizarov fixator. In ours work we try to find derotational mechanism that the most reduces adverse rotational and translational displacement of bone fragments and have the most accuracy of derotation. Optimal type of derotator will help to shorten treatment duration and improve treatment outcomes.

One of the main challenges faced by derotators is ensuring controlled derotation, even with large values of angular deformation, without having to change the configuration of the apparatus. Research on the physical model has shown that the studied derotators implement the assumed derotation with differing accuracy (Table 1). The observed variation is due to the specific structure of tested derotators and the amount of steps needed to complete one stage of derotation. *Z*-type derotator has a simple design, quick installation, and the possibility of correcting large torsional deformation. It is relatively easy to use by the patient, requiring three steps to perform derotation. Five tasks must be performed to use *H*-type derotator. In both cases, performing each step of derotation requires loosening the nuts of the derotator, which inevitably is the source of inaccuracy resulting from the implementation of applied correction. In the case of TR-type derotator, its design eliminates uncontrolled loosening of the mechanical system, which translates into the highest accuracy of the implementation of the projected aim of correction. Its disadvantage includes the possibility of correction torsional deformation in the range of up to 15°; gaining larger ranges requires modifying the structure of the fixator.

Physical model tests revealed a significant problem in terms of translational displacement of fragments accompanying rotation of the fragments (Table 1). In our opinion, the cause of this situation consists of two factors. The first is related to the asymmetry of the construction of the fixator. In each of the fixators three derotators were used, spaced every 120° around the circumference of the rings. The asymmetric distribution of Kirschner wires, taking into account the location of the safe zones of their introduction, causes an asymmetry in the mechanical structure of the fixator. The second factor results from the already discussed loosening occurring in the kinematic pairs of the derotator during the implementation of each degree of derotation. At that time, the internal stresses acting within the “fixator-bone fragments” system are released and produce transverse displacement of fragments .

Unlike other external fixators, the Ilizarov system uses thin transosseous wires that allow stable yet dynamic fixation of the bone fragments. To prevent large interfragmentary movements, the wires in the Ilizarov system must be able to withstand the axial load of the bone fragments during weight bearing. Biomechanical parameters such as the wire angle, the amount of wire tension and the wire material have been defined as improving overall frame stiffness.^3^,^4^,^6^

Gessmann introduced modification made by roughening the wire-bolt interface, which results in improved holding capacity and wire stiffness.^6^ Design of modificated nut, which reduced uncontrolled loosening of the mechanical system during each step of derotation, will translate into the highest accuracy of the implementation of the projected aim of correction and decreased translational displacement of fragments.

It follows that an extremely important issue, decisive for the success of torsional deformation treatment, is the well-thought out selection of the configuration of the construction of the fixator and its axial, bending and torsional stiffness.

Based on previous research conducted by the authors,^13^ it can be seen that the type (*Z*, *H*, TR) of used derotator has little effect on the axial, bending, or torsional base stiffness of Ilizarov fixator structure. It is possible to replace different types of derotators with each other, without affecting the stiffness properties of the Ilizarov fixator.

In the study of physical models, translational displacement of fragments associated with derotation has been observed. Correct installation of the apparatus on the patient and properly performed derotation allows for limiting detrimental influence of translational displacements on regenerating bone tissue. To some extent, newly-formed blood vessels are able to rebuild, compensating for translational displacement. This allows obtaining valuable regenerating bone tissue during derotation.

Based on physical model, estimating translational displacement of fragments and accuracy of derotation, two basic conclusions can be made. First, use of different derotators, at 15° derotation, has statistically significant impact on translational displacement, which can create different biomechanical conditions for the formation of the regenerating bone tissue. The translational displacements differences for the 30° derotation were not significant. Second, significant of different accuracy of derotation of the fixators with different derotation mechanisms suggest their unequal applicability.

Base on the translational displacement of fragments and accuracy of derotation, we can attempt to make some application preferences for the analysed derotators for the 30° derotation. For correction of rotational deformities up to 30°, it is advisable to use the type *Z* derotators due to its higher accuracy of derotation. Unfortunately, we cannot attempt to make any application preferences for the analysed derotators in the range of up to 15° configuration. During derotation, small translational displacement of fragments and high accuracy of derotation, are both important for successful treatment. The TR-type derotators had highest accuracy of the implementation of the projected aim of correction, but also had highest translational displacement. The *H*-type derotators had lowest translational displacement, but also had lowest accuracy of the implementation of the projected aim of correction.

Different types of derotators can affect the biomechanical conditions in the regenerating bone tissue through different kinematics characteristics.
